# Importancia de las características clínicas y de laboratorio en el diagnóstico de las infecciones concomitantes por dengue y chikungunya: reporte de un caso probable

**DOI:** 10.7705/biomedica.5085

**Published:** 2020-06-30

**Authors:** Berta Nelly Restrepo, Margarita Arboleda, Katerine Marín, Paola Romero, Ana Luisa Muñoz, Irene Bosch

**Affiliations:** 1 Instituto Colombiano de Medicina Tropical-Universidad CES, Sabaneta, Colombia Instituto Colombiano de Medicina Tropical-Universidad CES Sabaneta Colombia; 2 Instituto Colombiano de Medicina Tropical-Universidad CES, Apartadó, Colombia Instituto Colombiano de Medicina Tropical-Universidad CES Apartadó Colombia; 3 Grupo de Ingeniería de Biología Celular y Funcional y Biomoléculas, Facultad de Ciencias, Universidad Antonio Nariño, Bogotá, D.C., Colombia Universidad Antonio Nariño Grupo de Ingeniería de Biología Celular y Funcional y Biomoléculas Universidad Antonio Nariño BogotáD.C Colombia; 4 Institute for Medical Engineering and Science, Massachusetts Institute of Technology, Cambridge, United States of América Institute for Medical Engineering and Science Massachusetts Institute of Technology Cambridge United States of América

**Keywords:** fiebre chikungunya, dengue, coinfección, artralgia, hiperpigmentación, Colombia, Chikungunya fever, dengue, coinfection, arthralgia, hyperpigmentation, Colombia

## Abstract

Se presenta el caso de una mujer de 38 años que consultó inicialmente por fiebre indiferenciada. A pesar de que el cuadro clínico evolucionó con manifestaciones clínicas de dengue con signos de alarma y de que la detección de IgM antidengue en una sola muestra indicaba que se trataba de un caso probable que había podido ocurrir durante los tres meses anteriores, la paciente consultó de forma reiterada, pues no presentaba una mejoría significativa.

En el décimo día del inicio de los síntomas, se observó edema simétrico en múltiples articulaciones acompañado de dolor, así como lesiones hiperpigmentadas en el surco nasogeniano. Se confirmó el diagnóstico de chikungunya por la presencia de anticuerpos IgM. Aunque puede pasar desapercibida, en los países endémicos para dengue y chikungunya existe la posibilidad de la infección concomitante, la cual puede agravar la evolución clínica de cada una de estas enfermedades. Por ello, es necesario que el médico considere las características clínicas y de laboratorio de ambas enfermedades para diagnosticar su presencia simultánea, garantizar un manejo adecuado y minimizar las complicaciones.

## Abstract (en)

We report the case of a 38-year-old woman who initially consulted for an undifferentiated fever. Although her clinical condition evolved with signs and symptoms compatible with dengue with alarm signs and that the anti-dengue IgM detection in a single sample indicated it was a probable case that could have happened during the previous three months, the patient kept consulting due to little improvement.

On the tenth day after the onset of symptoms, she presented with painful polyarticular symmetric edema, as well as hyperpigmented lesions in the nasolabial fold. Chikungunya diagnosis was confirmed by the presence of IgM antibodies. In endemic countries for dengue and chikungunya, the possibility of co-infection exists, but it may go unnoticed. On the other hand, the co-infection may worsen the clinical course of these diseases. Therefore, physicians should evaluate the clinical and laboratory characteristics of both infections to be able to diagnose the coinfection for adequate management and to minimize complications.

El virus del dengue (DENV) y el de chikungunya (CHIKV) son transmitidos por la picadura de mosquitos infectados del género *Aedes*. El DENV pertenece al género *Flavivirus*, familia Flaviviridae y el CHIKV al género *Alphavirus*, familia Togaviridae ^1^.

Ambas arbovirosis son de importancia en salud pública en los países tropicales y subtropicales. Entre el 2010 y el 2016 se reportaron en Colombia 674.000 casos de dengue, y entre el 2014 y el 2016 se notificaron 774.831 casos de chikungunya ^2^.

La circulación de estos arbovirus en territorios con presencia de *Aedes aegypti* ha facilitado la aparición de infecciones concomitantes y, por ende, la intensificación de sus complicaciones, que pueden llegar incluso a ser mortales ^3^^-^^5^. Sin embargo, este diagnóstico es difícil, en parte porque las manifestaciones clínicas pueden superponerse, en tanto que las pruebas confirmatorias de laboratorio se recomiendan para los grupos de riesgo y la perspicacia clínica en su diagnóstico disminuye después de las epidemias. Por ello, la presunción diagnóstica basada en las manifestaciones clínicas y en los hallazgos de laboratorio es indispensable.

En el presente reporte de caso, se describen las manifestaciones clínicas y los hallazgos de laboratorio que permitieron el diagnóstico simultáneo de estas dos infecciones con el propósito de sensibilizar al personal de salud sobre su diagnóstico clínico temprano para brindar al paciente un manejo más adecuado y evitar las complicaciones.

## Reporte de caso

Se trata de una mujer de 38 años, residente en el municipio de Apartadó, Antioquia, que consultó el 2 de abril de 2018 por presentar cefalea, astenia, adinamia, sensación subjetiva de fiebre, náuseas y vómito, de un día de evolución. Se le trató ambulatoriamente con analgésicos y recomendaciones sobre los signos de alarma.

Al quinto día del inicio de los síntomas, la paciente consultó de nuevo porque continuaba presentándolos, además de mialgias, artralgias, exantema, prurito, dolor abdominal, inyección conjuntival y mareos, por lo que fue hospitalizada en el hospital de segundo nivel de atención.

En el examen físico se observó un brote petequial del tipo de “islas blancas en el mar rojo”. En los exámenes de laboratorio se evidenciaron trombocitopenia, leucopenia, hemoconcentración y anormalidades en las pruebas hepáticas (cuadro 1). Mediante ecografía, se encontró hepatoesplenomegalia y distensión fisiológica de la vesícula biliar. La presencia de IgM anti-DENV se verificó mediante una prueba comercial (Standard Diagnosis™, Abbot) en una sola muestra de suero, con lo que se consideró como un caso probable de dengue.

**Cuadro 1:** Evolución de los hallazgos de laboratorio en una paciente con infección concomitante por dengue y chikungunya

Hallazgos de laboratorio	**Días después de la fecha de inicio de los síntomas**							
	5°	6°	7°	10°	11°	12°	13°	17°
Hematocrito (%)	41,1	51,1	49,4	37,5	43,9	-	38,9	37,0
Hemoglobina (g/dl)	15	15	14,5	13,3	12,4	-	11,6	12,6
Plaquetas (células/10^9^/L)	93	93	113	184	306	-	292	389
Leucocitos (células/mm^3^)	2.800	-	4.200	4.520	5.200	-	6.600	-
Neutrófilos (células/mm^3^)	1.400	-	2.700	2.214	2.800	-	4.100	-
Linfocitos (células/mm^3^)	1.100	-	1.000	1.853	1.900	-	2.000	
AST(U/L)	-	116,7	-	109	71	-	36	18,9
ALT(U/L)	-	112	-	195	150	-	103	52,0
Bilirrubina total (mg/dl)	-	0,71	-	0,42	0,54	-	0,56	0,24
Bilirrubina directa (mg/dl)	-	0,29	-	0,31	0,42	-	0,47	0,09
Bilirrubina indirecta (mg/dl)	-	0,42	-	-	0,12	-	-	0,15
CPK (UI/L)	-	-	-	-	-	110	-	-

Rangos normales: hemoglobina: 12-17 g/dl; hematocrito: 40-50 %; plaquetas: 150-600/10^9^/L; leucocitos totales: 4,000-12,000 células/mm^3^; neutrófilos: 3,500-11,000 células/mm^3^; linfocitos: 1,300- 4,000 células/mm^3^; aspartato-aminotransferasa (AST): 10-34 U/L; alanino-aminotransferasa (ALT): 5-59 U/L; bilirrubina total: 0,3-1,9 mg/dl; bilirrubina directa: 0-3 mg/dl; bilirrubina indirecta: menos de 1,0 mg/dl; creatinina cinasa (CPK): 0 y 170 UI/L

A la paciente se le dio de alta al tercer día de la hospitalización y, diez días después del inicio de los síntomas, consultó de nuevo porque el malestar general y el prurito persistían. Se la hospitalizó nuevamente por presunción diagnóstica de infección concomitante de dengue y leptospira, ya que la paciente provenía de una zona endémica para esta; con base en tal presunción, el médico tratante prescribió tratamiento con penicilina cristalina y doxiciclina.

Durante esa segunda hospitalización, la paciente firmó el consentimiento informado para ingresar al estudio sobre la “Dinámica clínica, inmunológica y viral de la infección por chikungunya”, proyecto aprobado por el Comité de Bioética de la Universidad CES (Acta 82).

**Figura 1:**
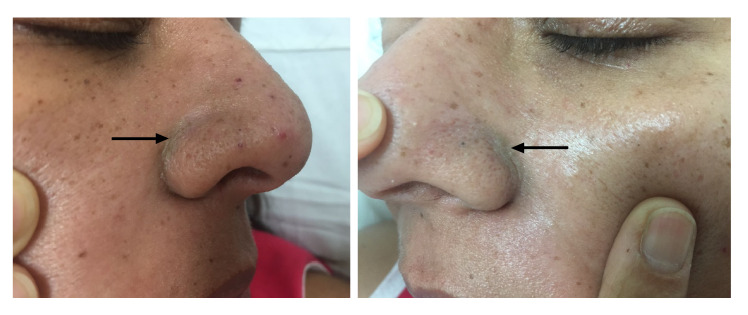
Hiperpigmentación en el surco nasogeniano en el décimo día del inicio de síntomas

Los síntomas persistieron después de una semana de evolución. En ese momento, se observó en el examen físico hiperpigmentación bilateral en el surco nasogeniano (figura 1), adenomegalias dolorosas en la región suboccipital y en la inguinal, y edema y dolor en las articulaciones de manos y pies, síntomas no descritos previamente en su historia clínica. En la prueba de detección de anticuerpos IgM (Novalisa™), se confirmó el diagnóstico de infección por chikungunya; se hizo, además, una PCR en tiempo real (RT- PCR) para chikungunya ^6^ y una para dengue ^7^^,^^8^, con resultados negativos.

A los cuatro días de esta segunda hospitalización, se le dio de alta ante la mejoría en los resultados de los exámenes de laboratorio. A los 17 días de los síntomas iniciales, la paciente volvió a consultar en un hospital de tercer nivel por dolor en la pantorrilla de dos días de evolución; se decidió hospitalizarla dados sus antecedentes, pero a los dos días se le dio de alta, pues su evolución clínica y los resultados de laboratorio fueron satisfactorios (cuadro 1).

Como parte del protocolo del estudio citado, el seguimiento de la paciente incluyó el grado de actividad inflamatoria mediante la evaluación del dolor medido con la escala visual análoga, y la presencia de dolor e inflamación en las articulaciones y del grado de discapacidad medidos por el *Health Assessment Questionnaire* (HAQ) ^9^, con los siguientes resultados.

En la fase aguda de la enfermedad (a diez días de los primeros síntomas), la paciente calificó el dolor con 10 puntos (máximo dolor); las articulaciones comprometidas fueron las de muñecas, tobillos y falanges de manos y pies, en las que refirió dolor y edema (artritis), y la cadera, en la que refirió solo dolor (artralgia). Presentó un grado de dificultad moderada para realizar actividades como bañarse, sentarse en una silla sin brazos, partir la carne con cuchillo, llevarse un vaso lleno a la boca, abrir tarros y subir escalas, y para algunas de ellas requirió la ayuda de otras personas. A los 30 días de los primeros síntomas (fase subaguda), calificó el dolor con 5 puntos en las mismas articulaciones, y refirió dolor y edema en los tobillos. El grado de dificultad para las actividades ya descritas fue leve.

En la evaluación a los tres meses de los primeros síntomas (fase crónica), la paciente calificó el grado de dolor aún con 5, pero solo en la cadera y las muñecas, y no presentó edemas. Relató, además, caída abundante del cabello. En la fase subaguda fue tratada por el reumatólogo con antiinflamatorios no esteroideos con lo que sus síntomas articulares mostraron mejoría.

Un mes después de los primeros síntomas, se descartó el diagnóstico de leptospirosis mediante la prueba de inmunofluorescencia indirecta (IFI), en la cual tanto los anticuerpos IgM como los IgG fueron negativos. El establecimiento de un diagnóstico adecuado fue clave para ajustar el tratamiento del caso.

## Discusión

La circulación de los virus de chikungunya y dengue en zonas con presencia de *A. aegypti* ha facilitado la aparición de infecciones concomitantes cuya frecuencia oscila entre el 0,01 y el 38 % en pacientes con síndrome febril ^4^^,^^5^^,^^10^^-^^18^. En estos estudios se incluyeron 28.604 pacientes (entre 24 y 23.871 pacientes por estudio), y la presencia de infección simultánea con CHIKV-DENV se confirmó en el 0,9 % de los casos (214 casos). Las diferencias de estas frecuencias se explicarían por el momento epidemiológico, el tamaño de la muestra y el tipo de técnica diagnóstica utilizada.

La infección concomitante con estos dos arbovirus se ha relacionado con una mayor gravedad de las manifestaciones clínicas. En una serie de 25 casos, la mortalidad fue significativamente mayor en los pacientes con infección concomitante ^4^. Otros estudios no comparativos describen hallazgos tales como el compromiso del sistema nervioso central en dos de los seis casos de infección concomitante ^5^, el desarrollo del síndrome de Guillain-Barré en tres de los pacientes con infección concomitante en estudio ^11^, la aparición de púrpura trombocitopénica trombótica ^19^ e, incluso, la muerte de un paciente por falla renal ^3^ y de varios más por otros motivos ^18^. En Colombia, un estudio *post mortem* de siete casos de infección simultánea por dengue y chikungunya evidenció el compromiso renal y hepático por estos dos arbovirus en la histopatología ^20^. Por el contrario, otros autores han descrito una evolución benigna en los casos de infección concomitante ^12^^,^^16^^,^^17^^,^^21^.

Al comparar las manifestaciones clínicas y los resultados de laboratorio de los pacientes con infección simultánea por los virus CHIKV y DENV, y los de aquellos con infección con uno solo de los virus, se ha encontrado que el dolor abdominal, el vómito, la cefalea y las manifestaciones hemorrágicas son significativamente más frecuentes en los pacientes con dengue o con la infección concomitante que en aquellos infectados únicamente con el CHIKV ^14^. Otros autores han observado una mayor frecuencia de trombocitopenia en los casos de infección solo por dengue y de infección simultánea con CHIKV y DENV que en los casos infectados solo por chikungunya ^22^. Además, los pacientes con infección concomitante por CHIKV y DENV referían dolor con un mayor puntaje en la escala visual análoga, así como mayor intensidad de las artralgias y mayor limitación de los movimientos de las articulaciones en comparación con los pacientes con diagnóstico solo de dengue ^13^.

Las manifestaciones en la piel son frecuentes en ambas infecciones. En los pacientes con dengue oscilan entre el 50 y el 82 % y, en aquellos con infección por chikungunya, están en cerca del 70 % ^23^^,^^24^. La manifestación cutánea más común en el dengue es la erupción máculo-papular y la morbiliforme que, al presentarse juntas, dan la apariencia de “islas blancas en un mar rojo”; también, se presentan la erupción petequial y las lesiones purpúricas ^25^^,^^26^.

En la infección por chikungunya, se ha descrito erupción morbiliforme, eritema macular, bulas y vesículas, úlceras, cambios en las uñas, hiperpigmentación y exacerbación de las dermatosis existentes ^27^^-^^29^; además, algunos autores han registrado la hiperpigmentación como el hallazgo más frecuente ^29^^,^^30^. La lesión puede aparecer en forma de máculas o pecas, pero también como melasma, melanosis periorbital o con un patrón de pigmentación flagelado. La ubicación más frecuente es la parte frontal de la cara en forma simétrica, aunque cualquier parte del cuerpo puede verse comprometida; puede aparecer durante la defervescencia o en la fase subaguda y persistir de tres a seis meses ^31^^,^^32^ y su causa se desconoce.

En la biopsia de estas lesiones, se observa una capa basal intacta con hipermelanosis difusa de toda la epidermis, sugestiva de un aumento de la dispersión de la melanina intraepidérmica y la retención del virus ^31^. En Colombia, se ha documentado poco este hallazgo dermatológico.

En el presente caso, las manifestaciones concordaron con lo descrito en los artículos citados en cuanto a la superposición de síntomas y los hallazgos de laboratorio en pacientes con infección simultánea por estos dos arbovirus ^14^^,^^22^^,^^25^^,^^26^. Sin embargo, en el inicio de la enfermedad, las hemorragias y los hallazgos de laboratorio (trombocitopenia, leucopenia y hemoconcentración), poco usuales en pacientes con infección por chikungunya, inclinaron la balanza hacia el diagnóstico de dengue con signos de alarma. A pesar del tratamiento adecuado, la paciente continuó sintomática y con compromiso hepático, lo que la llevó a consultar reiteradamente, aunque no se indagó por otros hallazgos clínicos que aumentaran la posibilidad del diagnóstico de infección concomitante, como la presencia de artritis o artralgias de pequeñas articulaciones en forma simétrica y de hiperpigmentación.

La infección concomitante con DENV y CHIKV se confirma mediante pruebas moleculares (RT-PCR) que permiten demostrar la presencia simultánea de los dos virus en el paciente. Su detección por la presencia de anticuerpos IgM tiene la desventaja de que, dado que estos anticuerpos son detectables en sangre hasta durante tres meses, la infección por uno de los virus puede haber precedido al otro, pero sin estar presentes simultáneamente. En algunos de los artículos consultados, los casos de infección concomitante de DENV y CHIKV se confirmaron con pruebas moleculares ^5^^,^^10^^,^^15^^,^^19^, en otros, solo con la detección de anticuerpos IgM ^4^^,^^11^^,^^17^, y en otros, mediante la combinación de ambas pruebas ^12^^-^^14^.

En cuanto al presente caso, la detección de anticuerpos IgM anti-DENV y anti-CHIKV, conjuntamente con las manifestaciones clínicas sugestivas de dengue y fiebre de chikungunya, permitieron concluir que la paciente presentaba una probable infección simultánea por CHIKV y DENV.

En los países endémicos para dengue y chikungunya, existe la posibilidad de la infección concomitante, aunque puede pasar desapercibida. Por otra parte, se ha observado que su presencia puede agravar la evolución clínica de estas enfermedades, por lo que los médicos tratantes deben estar atentos a las manifestaciones clínicas y a los resultados de laboratorio para ayudarse en el diagnóstico de la infección concomitante, con el fin de conocer su frecuencia y su perfil clínico y epidemiológico.
